# Time-sensitive therapeutics

**DOI:** 10.1186/s13054-017-1911-y

**Published:** 2017-12-28

**Authors:** John J. Marini

**Affiliations:** University of Minnesota, Minneapolis, MN USA

**Keywords:** Adaptation, Timing, Homeostasis, Bio-rhythms, Circadian, Stages of illness

## Abstract

Much of what we now do in Critical Care carries an air of urgency, a pressing need to discover and act, with priorities biased toward a reactive response. However, efficacy often depends not simply upon what we do, but rather on whether, when, and how persistently we intervene. The practice of medicine is based upon diagnosis, integration of multiple sources of information, keen judgment, and appropriate intervention. Timing may not be everything, as the well-known adage suggests, but in the intensive care unit (ICU) timing issues clearly deserve more attention than they are currently given. Successfully or not, the patient is continually attempting to adapt and re-adjust to acute illness, and this adaptive  process takes time. Knowing that much of what we do carries potential for unintended harm as well as benefit, the trick is to decide whether the patient is winning or losing the adaptive struggle and whether we can help. Costs of modern ICU care is enormous and the trend line shows no encouraging sign of moderation. To sharpen our effectiveness, reduce hazard, and pare cost we must learn to time our interventions, help the patient adapt, and at times withhold treatment rather than jump in on the impulse to rescue and/or to alter the natural course of disease. Indeed, much of the progress made in our discipline has resulted both from timely intervention when called for and avoidance or moderation of hazardous treatments when not. Time-sensitive ICU therapeutics requires awareness of trends in key parameters, respect for adaptive chronobiology, level-headed evaluation of the need to intervene, and awareness of the costs of disrupting a potentially constructive natural response to illness.

## Background

Intensivists have become adept in caring for critically ill patients and now enable many to survive illnesses that in prior years would have proven fatal. Improved survival has resulted not only from better understanding of individual diseases and implementation of useful innovations, but also from optimizing intensive care unit (ICU) organization, standardizing best practices, and improving key processes of care delivery. This decline in short-term mortality is a major achievement, but there is increasing awareness that chronic critical illness often continues well beyond ICU discharge, often culminating in long-term morbidity and mortality [1]. Why does this happen? The traditional principles of applied physiology provide the foundation upon which personalization and optimization of critical care are currently based. While these serve well during the rescue phase of intensive care, it is the thesis of this paper that our current knowledge of the *physiology of critical illness* is at a rudimentary stage and that we know relatively little about the continuously interactive processes—both natural and iatrogenic—that determine either an ultimately catastrophic outcome or appropriately adaptive response to the challenges of critical illness (Table 1).

**Table 1:** Patho-physiology of critical illness continually evolves

• Almost all treatments hold potential for injury to both targeted and untargeted organs. • Selection of treatment, dose, and duration should ideally be based on awareness of underlying dynamism of evolving pathophysiology. • *Thesis:* Well-intentioned treatments often frustrate and delay an appropriate adaptive response.	

Almost all treatments that we provide to the critically ill patient hold potential for injury to both targeted and non-targeted organs. Ideally, selection of treatment, dose, and duration should be based on awareness of the underlying dynamics of the evolving pathophysiology. It can be reasonably argued that well-intentioned treatments often frustrate and delay an appropriate adaptive response. Moreover, innate responses of the body to critical illness may themselves be inappropriate. Whereas it is an unassailable fact that homeostatic regulation is indispensable during health and moderate illnesses, the same may not be true in the presence of overwhelming challenge.

In his famous book “The Wisdom of the Body”, Walter B. Cannon outlined the intricate feedback mechanisms which allow and modulate appropriate responses to challenges to homeostasis [2]. He and others called attention to the intricacies of innate biorhythms which during health maintain an exquisite balance. Critical illness and treatments disrupt normal physiology and adaptive mechanisms, and often ignore biorhythms, destabilizing and perhaps invalidating normal physiological controls. Increasing evidence indicates that the body does not remain invariably “wise” during catastrophic illness.

Evolution may not have provided for appropriate responses to severe acute injuries. Until recent decades, such illnesses were not survivable. Indeed, to strengthen the gene pool, evolutionary pressures may have been biased toward ensuring an adverse outcome for susceptible individuals. In other words, evolved responses to life-threatening stresses might not be on side. The exuberant “rogue inflammation” response to a septic challenge provides one good example of how an exaggerated, counterproductive reaction may provoke or promote organ damage [3].

Enumerating the key characteristics of health and disease underlines the importance of time-based physiology to the expression and resolution of critical illness (Table 2). Pattern variation, appropriate corrections in response to moderate stress (allostasis), and diurnal biorhythms are expressions of adequate strength and endurance potential. During life-threatening critical illness these are replaced by the pattern rigidity, disproportionate reactions, and monotony that indicate loss of compensatory reserve [4, 5]. In health and in response to tolerable illness, gradual transitions prevail and homeostatic adaptation is expressed in response to stressors, whether mechanical, environmental, or biochemical. In severe disease, transitions are abrupt and there is a failure to adapt appropriately to the imposed stressor. Such inflexibility is often coupled to dysfunctionally exuberant or inadequate responses.

**Table 2:** Key characteristics of health and disease

Health	Critical illness
Variation	Rigidity
Homeostasis	Disproportionate reactions
Diurnal biorhythms	Monotony
Adaptability	Loss of adaptive reserve

Our medical job is to help the patient recover adaptive homeostatic control. In order to do this, the critical caregiver should aim to first attenuate dysfunctional early responses and then promote gradually adaptive homeostatic ones. To accomplish these goals, good intervention timing and dosing are essential (Table 3). Adaptive accommodation to a seriously stressful challenge often takes time to fully develop. A good example is provided by the dynamics of the heat shock response. After exposure to a brief but strong heating stress pulse, the synthesis of cell protective heat shock proteins is initiated quickly but only peaks many hours later [6]. Once fully developed, this  protection mitigates the damage resulting from a potentially injurious pattern of mechanical ventilation [7]. On the other hand, heating encountered synchronously with a similar injury-provoking ventilation stimulus markedly accentuates the deterioration of lung mechanics, oxygen exchange, and tissue injury [8].

**Table 3:** Timing issues in critical illness

• Stage of disease and recovery • Intensity of management • Length of application • Adaptation • Diurnal physiology	

Although the underlying and continuously evolving patterns of injury and response usually take place below the threshold of our clinical recognition, our therapeutic interventions influence the eventual outcome due to poorly timed imposition, maintenance, or withdrawal of treatment. Foremost among those that have received recent attention are excessive sedation and enforced bed rest for prolonged periods [9]. Undoubtedly there are others; in fact, I strongly believe that many of our current practices that encourage monotony (e.g., volume controlled ventilation, sustained drug infusions and feedings) or squelch variation (e.g., unnecessarily rigid targeting of isolated hemodynamic variables such as blood pressure) are counterproductive to long-term adaptive response.

In critical care, imprecise definitions and the impersonal approaches of randomized trials threaten to oversimplify management and encourage neglect of personalized physiologic dynamics. Randomized clinical trials, though often instructive and useful for hypothesis generation, often guide decision-making with answers that are interpreted to be ‘all or none’ categorical directives suitable for encoding into care protocols. Although generally helpful for treating the targeted population at large, at times these approaches may conflict with optimized care for the individual. Following such population-based ‘answers’, many critical care practitioners consider low tidal volumes to be appropriate for everyone [10], conservative fluid therapy invariably to be superior to liberal administration at all phases of acute respiratory distress syndrome (ARDS) [11], steroids to be inappropriate for all stages and forms of lung injury [12], etc. In reality, few practice-altering trials have been designed with deep and detailed understanding of the underlying mechanisms or account for individual variation, complexity, biological variation, and the timing of pathophysiology and treatment effects. Our current management approaches can be viewed as rather inflexible and primarily reactive management when, in fact, improved patient health demands proactive, time sensitive, and flexible strategies. The four Ds of drug, dose, duration, and de-escalation are applicable to many ICU interventions, including fluid therapy, antibiotics, and ventilatory support [13]. When facing a complex and evolving problem, the clinician requires appropriate tools, functional probes, and careful reasoning. The need for midcourse corrections should be anticipated and frequently made in response to monitored observations or relevant variables. These decisions must be rooted in physiological understanding. Sadly, however, that educational foundation and skill set has been seriously eroded by the electronically aided, “look it up” medical management structures in which we now work [14].

## Timing issues in critical illness

Precise and personalized critical care management requires awareness of certain timing issues that are often neglected. The critically ill patient passes through stages of disease and recovery which demand differing intensities of therapeutic intervention as well as keen awareness of when to withdraw external supports so as to allow adaptation and re-establishment of diurnal homeostatic physiology. The stages of critical illness can be viewed as progressing from rescue to stabilization, strengthening, and recovery. Wound healing progresses along such a timeline [15], and increasingly we are paying attention to the facts that pathologic expression varies widely among patients and that reactions to treatments continually evolve and change. We have been relatively slow to learn that responsiveness to many interventions depends on the stage of illness. In sepsis, immediate intervention with appropriate antibiotics is a key to survival, whereas prioritizing abrupt and aggressive fluid resuscitation may be somewhat less helpful [16]. Regarding ARDS, these stage-dependent interventions include positive end-expiratory pressure (PEEP) [17], prone positioning [18], recruitment maneuvers [19], neuromuscular blockade [20], corticosteroids [21], fluid management [22], and undoubtedly other common interventions that we have not yet seriously questioned (Table 4). For example, the internal endocrine environment continuously evolves as the acute inflammation of sepsis and ARDS progresses into the chronic and recovery phases [23]. Considerable experimental evidence indicates that the stages of illness should dictate metabolic therapy as well [24], with appropriate nutritional support and gut microbiome health depending on the composition and the timing of component feedings [25].

**Table 4:** Responsiveness to many interventions for ARDS depends on the stage of illness

• Positive end-expiratory pressure (PEEP) • Prone positioning • Recruitment • Neuromuscular blockade • Steroids • Nutrition	

The intensity issue is undoubtedly important but frequently ignored. For example, minute ventilation can be considered an intensity variable that determines whether an identical driving pressure for ventilation may cause injury or be well tolerated. The total power that lung tissue must endure is determined by the frequency of breathing as well as the conformation of the individual tidal cycle [26, 27]. The flow profile of each individual breath determines the rate at which alveolar pressure develops, and experimentally has been shown to be important in minimizing ventilator-induced lung injury [28, 29]. At the bedside, however, the inspiratory to expiratory ratio and inspiratory flow profile are given relatively little attention. Extending the duration of inspiration and ‘squaring’ the inspiratory flow profile have been shown in both small and large animal models to blunt the degree of injury inflicted by the same driving pressure. How fast strain is achieved is especially important when the lung is subjected to high stretching forces. In fact a recent experimental study suggests the driving airway or transpulmonary pressures—both based on static variables of plateau and PEEP—did not predict lung outcome when flow rate was altered through a wide range [30].

It is interesting to consider the question as to why early short-term muscle relaxants administered for a brief period early in ARDS demonstrated benefit which emerged much later with regard to mortality [20]. It is tempting to speculate that by attenuating the intensity of the initial native response we interrupt a catastrophic early feedback sequence which eventually would result in the patient’s demise. Along a similar vein, early sepsis intervention, though obviously important, sometimes may carry unintended consequences in situations where sudden cell lysis under the influence of antibiotics provokes inflammation and threatens survival [31]. Again, the unchecked exuberance of the body’s innate response may not always be helpful; this idea is given further support by demonstrations that early corticosteroids improve all-cause mortality in community-acquired pneumonia and blunt tendency for treatment failure [32]. In fact, early steroids appear to help stabilize severe pneumonia [33].

We have also learned harsh lessons regarding the appropriate length of application of our drugs and treatments. After the second phase of stabilization, decisions must be made regarding duration of treatment and the program for weaning support. It has been suggested that the reasons why corticosteroids hastened liberation from mechanical ventilator but failed to improve survival in the ARDS Network trial [34] are linked to inadequate duration of their use; in other words, steroids were stopped too soon. Perhaps the more common problem, however, is that we apply aggressive treatments for too long. It is clear that sustained steroid and neuromuscular blocking agents will weaken or atrophy muscle, producing ventilator-induced diaphragmatic dysfunction and peripheral muscle weakness that delay recovery [35, 36]. Excessive and long-term use of sedation is strongly suspected of contributing to delirium and sustained cognitive impairment in all age groups after critical illness. A link has been established between duration of delirium and long-term impairment of cognition [37]. Perhaps by using less sedation and fewer opiates we may mitigate this process.

One of the most important timing issues of critical illness concerns our interference with the body’s natural adaptive processes. The normal human body has an incredible capacity to adapt to stress. Endurance athletes have completed more than 50 marathons on consecutive days [38], high-altitude acclimatization has allowed multiple climbers to ascend Mount Everest without oxygen [39], and extraordinary adaptation to low temperature has been demonstrated by motivated and gradually trained individuals [40]. However, the capacity for the critically ill to adapt to the stresses of acute and subacute disease has not been extensively or systematically probed. Nevertheless, permissive hypercapnia [41] and more recently graded permissive hypoxemia [42] appear to offer well-tolerated alternatives to potentially noxious interventions such as high pressure ventilation and high inspired concentrations of oxygen. It has been argued that we should more aggressively encourage adaptation in the ICU by resetting our targets and gradually but methodically reloading the patient’s systems by graded withdrawal of supports required to sustain life during the initial days [43]. Such retargeting might be directed toward goals for blood pressure, hemoglobin, muscular workloads, and position, as well as blood gases. We know little about the advisability of imposing stress for brief periods in a fashion parallel to that of heat shock exposure. It has been shown, however, that adaptive ischemic preconditioning (intentional “stunning”) reduces infarct size in experimental coronary occlusion [44]. It is been suggested that inter-organ adaptive preconditioning (limb stress helping to condition other organs, for example) might also occur via hormonal or neural reflex pathways [45].

Were encouraging adaptation to critical illness a viable possibility, there would be a modified two-stage approach to management. The initial rescue phase would minimize demands, providing full support, encouraging gentle transitions and tolerance of monotonous supportive treatments such as continuous infusions and fully controlled mechanical ventilation. In the adaptation phase, there would be intermittent stresses in rest periods, with ongoing targeted reductions of vital supports to acclimatize the patient. These would include FiO_2_, ventilating pressure, vasopressors, and body position. Variability—not monotony—would be encouraged. Although, “ICU conditioning” is attractive in concept, major questions remain unanswered before such an approach can be advocated. These include: Are injured tissues capable of stress conditioning? Or are they hibernating or to injured to respond? Which variables should we monitor to guide the rate of withdrawal of life-sustaining measures? Can we rely on bedside biomarkers of distress and reserve? Can we automatically program or protocolize the graded withdrawal of support? Which conditioning pattern is optimal?

**Fig. 1:**
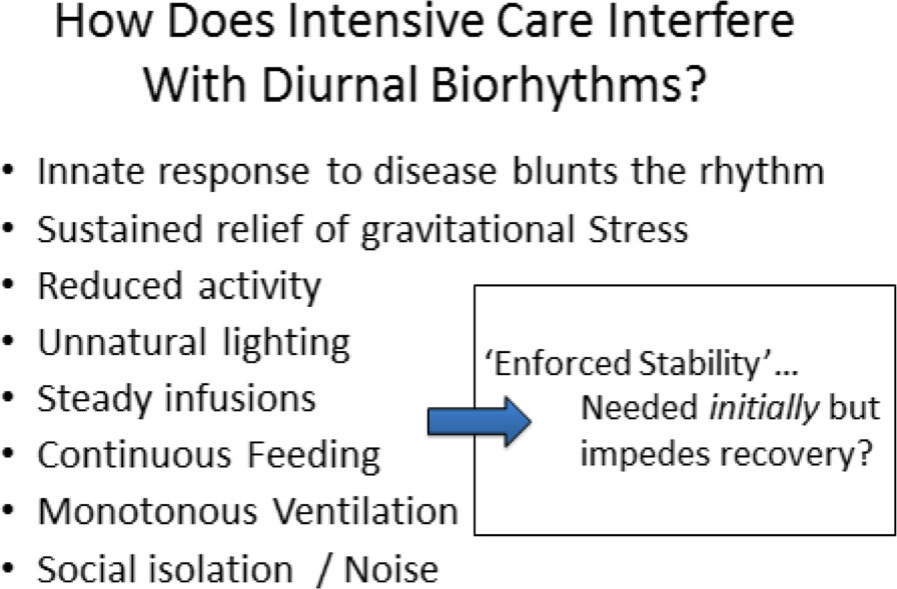
How does intensive care interfere with diurnal biorhythms?

An important but largely neglected aspect of our management of the critically ill relates to diurnal and circadian physiology [46] (Fig. 1). Although we are well aware of sleep-wake cycles, most practitioners are relatively oblivious to the brain organ crosstalk that may determine eventual outcome. Neural pathways and hormonal communications link many organs with the brain. Indeed, the potential for two-way neuroinflammatory linkage has been well described [47]. Recent reports regarding patient-ventilator asynchrony strongly suggest that important prognostic information may be gleaned from determining its incidence and clustering, and that ignoring the demands of the neural controller of the breathing pattern might even contribute to adverse outcomes through as yet undetermined pathways [48].

Most organ systems have some degree of brain-influenced circadian rhythm. The supra-chiasmatic nucleus (SCN), which itself is influenced by light exposure, motion, and other cues, is the master clock that regulates the peripheral clocks of other organ systems and sets the circadian rhythms of temperature, sleep-wake cycles, and metabolic, neuroendocrine, and cardiovascular regulations [49]. Melatonin appears to be central to such connections; its activity affects not only wakefulness but also endocrine function such as growth hormone and cortisol regulation, cardiovascular function in terms of heart rate variability and vascular tone, and immune cell function [50]. Melatonin strongly influences the inflammatory response via the antioxidant cascade, reducing oxidative stress when levels are high. The complexity of such interactions will require considerable additional research in the intensive care setting to determine the importance of maintaining appropriate diurnal biorhythms. Whatever the explanation, however, diurnal variation of inflammatory and oxidative sensitivity to lipopolysaccharide (LPS) has been shown in humans as well as experimental animals [51]. Presentation of LPS to rats at the wrong time of their diurnal cycle predisposes to severe injury or death, whereas animals challenged at the opposite time in the diurnal pattern show much greater tolerance.

The therapies that we apply in the ICU cause circadian dysrhythmias [52, 53]. The deleterious effects of noise, artificial light, stress, medical interventions, sedatives, and anesthetics interact with genetic predisposition to cause asynchrony. Innate response to disease blunts normal biorhythms, but we accentuate these tendencies with sustained relief of gravitational stress-reduced activity, steady infusions of drugs, continuous feedings, monotonous ventilation, social isolation, excessive noise, etc. Although this enforced stability may be needed initially, it likely impedes recovery when sustained. There are likely to be multiple contributors to diurnal biorhythm asynchrony. Critical illness alters the amplitude and variability of neuroendocrine hormones, a phenomenon which may contribute to an observed circadian incidence of cardiac arrhythmia such as ventricular tachycardia in critically ill patients, with a greater incidence during the day and lesser incidence at night. Considerable experimental evidence indicates that circadian disruption predisposes to cardiac arrhythmia [52] and disorders inflammatory responses [53]. Sleep deprivation, a well-recognized problem in critical care units, may itself blunt immune competence [54]. The role of circadian disruption in the generation of delirium has been recently explored by attempting to intervene by imposing diurnal light amplification [55]. Failure of light therapy alone to influence the incidence of delirium simply underscores that many factors contribute to this problem [56] and, as has already been mentioned, multiple factors apart from light exposure contribute to diurnal biorhythm patterns. Physical activity, auditory cues, and gravitational stresses may help re-establish appropriate diurnal physiology.

New approaches to understanding dynamic physiology and time-based therapeutics will require better matching of patient to treatment, better tracking of the evolution of the underlying physiology, carefully modulated intensity and duration of therapeutic interventions, attention to re-establishing natural biorhythms, and perhaps deliberate stress conditioning (Table 5). Although we currently lack suitable biomarkers, certain dynamic functional probes of patient capability have already been implemented. One example is the awake and breathe (ABC) trial in which an awakening intervention, followed by spontaneous breathing, showed better results than the conventional approach lacking the awakening component [57]. Clinicians have become adept at using certain bedside biomarkers such as brain natriuretic peptide (BNP), C-reactive protein (CRP), and procalcitonin. Indeed BNP may provide a good weanability indicator in well selected patients [58]. These humoral bio-markers, however, are not well suited to the moment by moment tracking of the patient’s underlying status with regard to the stabilization and recovery phases of illness. The bedside biomarkers of tomorrow, such as genomics, transcriptomics, proteomics, and metabolomics, offer both promise and limitation [59]. We currently lack suitable humoral biomarkers that pinpoint the stage of recovery. Associating detailed biochemical and physiologic information with newly developed technologies, however, may eventually disclose informative patterns of response. Certain physiological observations such as temperature pattern may eventually be integrated by “big data” analytics into important decision supports [60, 61]. Perhaps for the first time in history the complexity of continuously evolving molecular interactions may be monitored and trended to track the underlying dynamic physiology of critical illness. Such innovations point the way to time-sensitive individualized care throughout the continuum of life-threatening disease [62].

**Table 5:** New approaches to time sensitive dynamic physiology

• Precisely match patient to treatment – Gene arrays – Big data analytics – Selection – Trending of progress and response • Track the evolution of the underlying physiology – Functional monitoring – Follow trends of integrated variables – Selective biomarkers • Modulate intensity • Optimize duration	

## Summary

New approaches to time-sensitive dynamic physiology include better matching of patient to treatment, tracking the evolution of the underlying physiology with functional monitoring, following trends of integrated variables and selected biomarkers, and modulating the intensity and duration of our life supports. We need to restore circadian rhythms by providing the appropriate ambient environment, promoting activity and gravitational stress, and encouraging natural sleep-wake cycles by physical measures, perhaps aided by pharmacological adjuvants such as modafinil and melatonin. We require improved research methodologies that employ more biologically plausible disease models that allow study over extended periods so as to pursue our time-weighted research focus. We need to keep in mind a two-stage approach that stabilizes the early response and then encourages recovery of adaptive homeostasis. In doing so we may eventually flip the switch from reactive to better informed, time-sensitive, proactive therapeutics (Table 6).

**Table 6:** A two-stage approach to critical care

• Rescue phase – Minimize demand and establish stability – Full support/gentle transitions – Take control (monotony may be needed) • Adaptation phase – Intermittent stresses and rest periods – Ongoing targeted reductions of vital supports (acclimatize) – FiO_2_ – Ventilating pressure – Vasopressors – Positioning – Encourage variability	
